# Schizophrenia polygenic risk score and type 2 diabetes onset in older adults with no schizophrenia diagnosis

**DOI:** 10.1097/YPG.0000000000000349

**Published:** 2023-07-04

**Authors:** Diana Shamsutdinova, Olesya Ajnakina, Angus Roberts, Daniel Stahl

**Affiliations:** a Department of Biostatistics and Health Informatics, Institute of Psychiatry, Psychology and Neuroscience, King’s College London; b Department of Behavioural Science and Health, Institute of Epidemiology and Health Care, University College London, London, UK

**Keywords:** comorbidity, healthy ageing, polygenic risk score, schizophrenia, type 2 diabetes

## Abstract

**Objectives:**

An association between type 2 diabetes (T2DM) and schizophrenia has long been observed, and recent research revealed presence of shared genetic factors. However, epidemiological evidence was inconsistent, some reported insignificant contribution of genetic factors to T2DM-schizophrenia comorbidity. Prior works studied people with schizophrenia, particularly, antipsychotic-naive patients, or those during the first psychotic experience to limit schizophrenia-related environmental factors. In contrast, we controlled such factors by utilizing a general population sample of individuals undiagnosed with schizophrenia. We hypothesized that if schizophrenia genetics impact T2DM development and such impact is not fully mediated by schizophrenia-related environment, people with high polygenic schizophrenia risk would exhibit elevated T2DM incidence.

**Methods:**

Using a population-representative sample of adults aged ≥50 from English Longitudinal Study of Ageing (*n* = 5968, 493 T2DM cases, average follow-up 8.7 years), we investigated if schizophrenia polygenic risk score (PGS-SZ) is associated with T2DM onset. A proportional hazards model with interval censoring was adjusted for age and sex (Model 1), and age, sex, BMI, hypertension, cardiovascular diseases, exercise, smoking, depressive symptoms and T2DM polygenic risk score (Model 2). According to the power calculations, hazard rates > 1.14 per standard deviation in PGS-SZ could be detected.

**Results:**

We did not observe a significant association between PGS-SZ and T2DM incidence (hazard ratio 1.04; 95% CI 0.93–1.15; and 1.01, 95% CI 0.94–1.09).

**Conclusion:**

Our results suggest low contribution of the intrinsic biological mechanisms driven by the polygenic risk of schizophrenia on future T2DM onset. Further research is needed.

## Introduction

Schizophrenia is a highly heritable mental illness with a lifetime prevalence of 0.5–1% (Simeone *et al.*, 2015; Public Health England, 2018). It is associated with elevated rates of comorbid diseases and a four to 13 times higher mortality rate, leading to shorter life expectancy by up to 20 years (Lawrence *et al.*, 2013). People with schizophrenia are particularly susceptible to metabolic dysfunction and type 2 diabetes (T2DM), characterized by a persistently elevated blood glucose concentration. The prevalence of T2DM in schizophrenia can be as high as 10–30% (Das-Munshi *et al.*, 2017), which is two to three times higher than T2DM rate in the general population (Stubbs *et al.*, 2015). T2DM is among the main reasons for the excess mortality in schizophrenia (Suvisaari *et al.*, 2013), which reiterates the urgency to understand contributions of various risk factors to schizophrenia and T2DM comorbidity.

Previously, elevated rates of T2DM in adults with schizophrenia had been mainly attributed to the side effects of antipsychotics, however, present studies agree on a multifactorial nature of the relationship between schizophrenia and T2DM (Ward and Druss, 2015). Common T2DM risk factors, such as low physical activity, poor diet and low socio-economic status, are prevalent in people with schizophrenia and can be amplified by schizophrenia-related factors such as antipsychotics and cognitive impairments (Ward and Druss, 2015). In light of the growing evidence of metabolic changes in antipsychotic-naive patients and during the first episode of the illness (Pillinger *et al.*, 2017; Rajkumar *et al.*, 2017), the presence of the shared biological mechanisms has been investigated (Mizuki *et al.*, 2020). Supporting this hypothesis, twin and familial studies have found a considerable genetic component linking T2DM and schizophrenia (Sullivan *et al.*, 2003; Almgren *et al.*, 2011; Willemsen *et al.*, 2015).

The recent development of genetic methods such as genome-wide association studies (GWAS) has revealed a highly polygenic architecture of T2DM and schizophrenia, with many genetic variants contributing to both diseases (Lin and Shuldiner, 2010; Hackinger *et al.*, 2018). Building on GWAS results, polygenic scores (PGS) have emerged, which measure individual liability to a disorder. PGS are computed as a sum of common genetic variants weighted by log-odds of their effect sizes across the risk alleles identified by GWAS (Wray *et al.*, 2014); a high PGS means that a large number of variants associated with the disorder are found in the individual genotype. PGS for schizophrenia (PGS-SZ) were associated with adverse symptoms strength (Richards *et al.*, 2020), treatment-resistance (Frank *et al.*, 2015) and progression to schizophrenia (Vassos *et al.*, 2017), indicating the association of PGS-SZ with the severity of the disorder. Several studies have employed PGS to link schizophrenia polygenic risk to the risk of T2DM: PGS-SZ may predict insulin resistance (Tomasik *et al.*, 2019), inflammatory and metabolic alterations (Maj *et al.*, 2020) and poor glycaemic control (Cao *et al.*, 2017). Nonetheless, negative findings have also been reported. For example, no correlation was found between glucose control and PGS-SZ in nonaffective psychosis (Habtewold *et al.*, 2020).

Other genetic methods, such as Mendelian Randomization and Linkage Disequilibrium score regression (LDSR), were applied to investigate T2DM-SZ comorbidity. LDSR uses GWAS summary statistics to quantify contributions of polygenic effects and can estimate genetic correlations. Mendelian Randomization is a technique that uses known genetic variants linked to a disease to test causal relationships between a trait and the disease. Using LDSR, schizophrenia was found genetically correlated with glucose abnormalities and hip-to-waist ratio (So *et al.*, 2019), with fasting insulin levels (Li *et al.*, 2018) and an eating disorder anorexia nervosa (Bulik-Sullivan *et al.*, 2015). In contrast, other works reported no genetic overlap (Polimanti *et al.*, 2018), or a small negative genetic correlation between schizophrenia and T2DM (Perry *et al.*, 2022) and metabolic syndrome (Aoki *et al.*, 2022), while a positive correlation would be expected to link an increased T2DM risk and schizophrenia genetics. Mendelian Randomization analyses were also inconsistent: So and colleagues (So *et al*., 2019) demonstrated schizophrenia’s causal role in metabolic abnormalities such as raised triglycerides; others found none (Li *et al*., 2018; Polimanti *et al*., 2018; Aoki *et al*., 2022) in either direction (Polimanti *et al*., 2018; Aoki *et al*., 2022).

Noticeably, prior studies of T2DM-schizophrenia comorbidity have naturally been focused on people with schizophrenia. To control for schizophrenia-related environmental factors, many employed antipsychotic-naive patients (Kirkpatrick *et al.*, 2012; Greenhalgh *et al.*, 2017), or people experiencing their first psychotic episode (Zhang *et al.*, 2015; Pillinger *et al*., 2017; Steiner *et al.*, 2019), other adjusted for environmental factors such as economic status, level of education and medication (Annamalai *et al.*, 2017; Das-Munshi *et al*., 2017); however, schizophrenia-related factors such as low social support, lifestyle habits and psychological stress of psychotic experiences (Kirkpatrick *et al*., 2012; Ward and Druss, 2015; Kasteridis *et al.*, 2019; Pillinger *et al.*, 2020) may be difficult to control in a sample of affected people, either due to the absence of relevant information or due to the fact that most people with schizophrenia. In turn, it may bias estimates of the impact of the underlying biological mechanisms of schizophrenia on T2DM rates. Similar argument can be applied to any case–control study on this topic, including genetic research, as it would draw conclusions from an underlying assumption that cases have higher genetic predisposition to schizophrenia than controls. Although this is a reasonable assumption, it can be difficult to separate the genetic and environmental factors causing the differences between these groups, especially in the presence of gene-environment correlations (Abdellaoui *et al.*, 2022) and interactions (VanderWeele *et al.*, 2014).

**Fig. 1:**
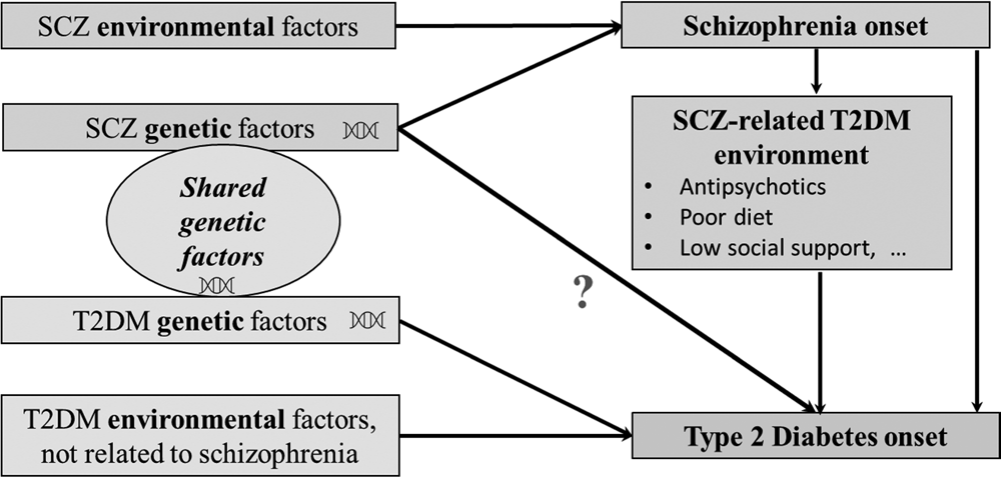
Risk factors involved in the association of type 2 diabetes and schizophrenia. The diagram is based on the three reviews (Lin and Shuldiner, 2010; Ward and Druss, 2015; Mizuki *et al*., 2020). The present study investigates whether the genetic predisposition to schizophrenia is associated with the risk of T2DM onset in the absence of schizophrenia diagnosis, utilizing a general population sample. TD2M, type 2 diabetes.

In light of the previously mixed findings, we aim to bring another evidence to the multifaceted T2DM-schizophrenia relationship. Instead of contrasting people with and without schizophrenia, we employ PGS-SZ to measure individual genetic susceptibility to schizophrenia in a general population sample of older adults (aged ≥50) with no schizophrenia diagnosis from the English Longitudinal Study of Ageing (ELSA) (Steptoe *et al.*, 2013), and fit a proportionate hazards model to measure the PGS-SZ- T2DM association with T2DM onset. We hypothesize that if schizophrenia genetic factors do impact T2DM development and are not entirely mediated by the above-mentioned environmental factors, people with high schizophrenia polygenetic risk would exhibit elevated T2DM incidence rates in the following 9 years (Fig. 1). An underlying mechanism could be that people with polygenic liability to schizophrenia inherit diabetogenic traits linked to schizophrenia, such as metabolic dysregulation (Aoki *et al*., 2022), inflammation (Tsalamandris *et al.*, 2019; Perry *et al*., 2022), sleeping patterns (Byrne *et al.*, 2016), or eating disorders (Bulik-Sullivan *et al*., 2015; Solmi *et al.*, 2019). An important advantage of our relatively simple regression analysis is the ability to quantify PGS-SZ’s impact on T2DM incidence with limited bias, while advanced genetic methods such as Mendelian Randomization which were argued to be beneficial in identifying the presence of an association rather than measuring its strength (VanderWeele *et al*., 2014). Indeed, the general population sample naturally limits environmental factors associated with schizophrenia including treatments’ side effects; while the sample’s representativeness allows for assessing associations across a population-wide range of genotypes.

## Methods

### Sample

The sample came from the large and well phenotyped ELSA dataset (Steptoe *et al*., 2013). ELSA is an ongoing multidisciplinary study developed by a team of researchers based at University College London, the Institute for Fiscal Studies, and the National Centre for Social Research. The core ELSA cohort was recruited in 1998–2000 and included 11391 individuals aged ≥50, representative of the older noninstitutionalized UK population (Steptoe *et al*., 2013). Additional participants were invited at later stages to maintain representative age distribution. The participants were followed biennially with questionnaires starting from wave 1 (2002/3). In addition, medical examinations, including blood tests, took place every four years at waves 2 (2004/5), 4 (2008/9), 6 (2012/13), 8 (2016/17) and 10 (2020/2023). In this project, we used the information from wave 2 (2004/5) to wave 8 (2016/17), which were the first and the last completed waves that involved medical examinations.

Ethical approval for each ELSA wave was granted by the National Research Ethics Service (London Multicentre Research Ethics Committee); all participants gave informed consent. The datasets analyzed during the current study are available in UK Data Services and can be accessed at https://discover.ukdataservice.ac.uk.

### Inclusion criteria

The baseline of our study was the time of participants’ first nurse visit when blood was first collected, which was wave 2 (2004/5 for 82% of the sample) or wave 4 (2008/9 for the remaining 18%), depending on an individual entry point. Initial diabetes status and all covariates were measured at that time. We included participants with the available genetic information, with no T2DM or schizophrenia diagnosis at the baseline, and the outcome measure available for at least one wave after the baseline. As PGSs are built on European ancestry GWAS, participants of non-European descent were excluded.

### Type 2 diabetes outcome

T2DM status was established by self-report and blood test results (Stringhini *et al.*, 2016; Aguayo *et al.*, 2019). Blood-based diagnosis was based on the glycated haemoglobin (HBA1c) level, using a threshold of HbA1C ≥ 48 mmol/mol (6.5%) (World Health Organization, 2011). Self-reported diabetes was coded from the respondent’s answers [“Has a doctor ever told you have (diabetes)”] and was previously validated (Pierce *et al.*, 2009). Although the questionnaires did not distinguish the type of diabetes, all cases were assumed to be T2DM given the participants were older than 50 (Demakakos *et al.*, 2012; Stringhini *et al*., 2016).

### Covariates

We included a range of known T2DM risk factors in the ELSA data (Nice, 2015). Age and BMI were entered as continuous variables; sex was categorical. BMI was calculated using the standard formula (kg/m^2^) from the weight and height measured during the medical visits. Self-reported history of hypertension, stroke and cardiovascular diseases was binary (yes/no). Cardiovascular diseases included self-reported prevalent diagnoses of angina, heart attack, myocardial infarction, congestive heart failure, heart murmur and an abnormal heart rhythm. HDL cholesterol (mmol/l) and triglycerides (mmol/l) from the baseline blood test were continuous. The presence of depressive symptoms was established by the 8-item version of the Centre for Epidemiologic Studies Depression Scale, found to be comparable to the full 20-item scale (Karim *et al.*, 2015); a score ≥ four defined participants with severe depressive symptoms (Hamer *et al.*, 2012). Behavioural characteristics included current smoking status and exercise regime. Smoking status was defined as a current smoker (“yes”) or a nonsmoker (“no”), which included current nonsmokers and those who had never smoked before the interview. Exercise regime was categorized as “vigorous” for vigorous exercise ≥1/week; “moderate” for moderate exercise ≥1/week; “low/none” otherwise, based on the self-reported exercise frequencies. Socio-economic status was represented by education level and accumulated wealth. Education level had three categories (0 – tertiary education, 1 – upper secondary and vocational training and 2 – less than lower secondary education) (Schneider, 2008) based on the education history. Wealth status was established from the collective value of the property, savings, investments and nonfinancial assets such as artwork and jewellery, net of debt and mortgages, which was then tertiled into the low/medium/high categories (Stringhini *et al*., 2016; Zaninotto and Steptoe, 2019). Finally, genetic ancestry and polygenic predisposition to schizophrenia and T2DM (see below) were included as covariates.

### Genetic data

The genetic data were extracted from blood samples taken during home visits. The genome-wide genotyping was performed at University College London Genomics in 2013–2014 using the Illumina HumanOmni2.5 BeadChips (HumanOmni2.5-4v1, HumanOmni2.5-8v1.3, Illumina Inc., San Diego, California, USA).

#### Quality control

Single-nucleotide polymorphisms were excluded if they were nonautosomal, the minor allele frequency was <1% if more than 2% of genotype data were missing, and if the Hardy–Weinberg Equilibrium *P* value<10^−4^. Samples were removed based on call rate (<0.99), suspected non-European ancestry, sex difference in allelic frequency of ≥0.2, heterozygosity and relatedness. Presence of the closely related individuals can violate the independence of observations assumption and may lead to biased results. To assess the relatedness, identical by descent probabilities were computed for each pair of participants using the method of moments implemented in PLINK 1.9 (Chang *et al.*, 2015). The probability of 1 represents duplicates or monozygotic twins, 0.5, 0.25 and 0.125 – first-, second- and third-degree relatives, with some variability due to genotyping error, linkage disequilibrium and population structure. Therefore, we excluded one individual at random from the pairs with the identical by descent probability above 0.2, which is halfway between the third- and second-degree relatives (Laurie *et al.*, 2010; Marees *et al.*, 2018). We further calculated principal components as measures of genetic ancestry, which then were used to adjust for possible remaining population stratification in the association analyses (Price *et al.*, 2006).

#### Polygenic score

Polygenic scores for schizophrenia (PGS-SZ) were computed based on the 2020 GWAS by the Schizophrenia Working Group of the Psychiatric Genomics Consortium (Ripke *et al.*, 2020), which was a combined meta-analysis of 69 369 individuals with a diagnosis of schizophrenia and 236 642 controls; polygenic scores for T2DM (PGS-T2DM) were based on the GWAS of the DIAbetes Genetics Replication study and Meta-analysis Consortium (Morris *et al.*, 2012). As previous research highlighted that PGSs built from directly genotyped data either had more predictive power (Okbay *et al.*, 2016) or did not differ significantly from PGSs calculated using imputed data (Ware *et al.*, 2017), we calculated PGSs based on genotyped data. PGSs were calculated as a weighted sum of the allele dosages, summing over the markers abiding by the *P* value threshold (*P*_T_) (i.e. 0.001, 0.01, 0.05, 0.1, 0.3 and 1) weighted according to the strength of effect estimate were summed in a continuous score using PRSice (Euesden *et al.*, 2015). As a large comparative study previously showed that a PGS at *P* value thresholds *P*_T_ = 1 was the ultimate PGS to use in longitudinal studies (Okbay *et al*., 2016; Ware *et al*., 2017), we utilized PGS-SZ and PGS-T2DM that were based on *P*_T_ = 1 assuming all genetic markers contribute to trait development (Ajnakina and Steptoe, 2020). To aid interpretability, PGSs were normalized to a mean of 0 and an SD of 1.

### Statistical methods

All statistical analyses were conducted in RStudio version 3.6.1 (Stekhoven and Bühlmann, 2012). All tests were two-tailed; *P* values ≤ 0.05 were considered statistically significant.

#### Missing data

Some covariates had missing data: BMI for 354 (5.9%) participants, smoking status for 24 (0.4%), education for 970 (16.3%) and triglycerides and HDL cholesterol for 398 (6.7%). As the representativeness of the initial sample can be impaired in complete cases analyses (Donders *et al.*, 2006), we performed multiple imputations with random forest (Doove *et al.*, 2014) implemented in the R package mice (van Buuren and Groothuis-Oudshoorn, 2011), which was shown to produce less biased estimates in health records compared to linear methods (Shah *et al.*, 2014). We generated 20 versions of the data and pooled the results using Rubin’s rule (Rubin, 1987). To assess imputation quality, we compared variables distribution before and after the imputation, which was similar.

#### Regression analysis

To estimate the association of the PGS-SZ and the outcome, we used a proportional hazards model with interval censoring in R package icenReg (Anderson-Bergman, 2017b). Interval censoring occurs when the outcome status is observed periodically, so only an interval when the event happened is known. The method accommodated the uncertainty of the T2DM onset between the data collection waves. In interval censoring, regression parameters are estimated by maximizing log-likelihood, which includes terms representing the probability of the event falling into an interval, as opposed to a specific timepoint, as in the Cox model (Cox, 1972). IcenReg was shown to achieve a faster and more reliable convergence than other interval censoring methods (Anderson-Bergman, 2017a). Due to an increasing number of missing covariates as the study progressed (up to 25% at the second medical assessment), the unfeasible computational time it would take to perform multiple imputations, as well as the absence of interval censoring methods handling time-variant covariates, only the baseline covariate values were used in the analysis; however, we tested how the results would differ if we update covariate values at the next follow-up and employ Cox model with time-variable covariates (details in the following Sensitivity analyses section).

We fitted two regression models to measure the strength of the PGS-SZ – T2DM onset association using different levels of adjustments. Model 1 included PGS-SZ, adjustments for genetic ancestry (four principal components), age and sex. Model 2 also accounted for BMI, prevalent hypertension, prevalent cardiovascular diseases, severe depressive symptoms, current smoking, exercise regime, level of education, accumulated wealth and PGS-T2DM. As this was an exploratory study, which does not strictly require adjustment for multiple comparisons (Bender and Lange, 2001), we did not employ correction for multiple testing.

#### Power calculations

The smaller the effect of a variable, the larger the sample size needed to detect it with a given probability (power) and a *P* value threshold. We computed a minimum effect size that can be detected in our sample with 0.80 power and a *P* value of 0.05 using the R package powerSurvEpi (Qiu *et al.*, 2021). For our sample size of 5968 with 493 events, hazard ratios (HRs) of 1.14 per SD change or higher are likely to be detected with 80% power and a *P* value threshold of 0.05 (Hsieh and Lavori, 2000).

#### Sensitivity analyses

First, a complete case analysis was performed to control the impact of missing data imputation. Second, our sample included people older than 50, and participants with higher PGS-SZ could have already been diagnosed with T2DM at inception, and our analysis may have included “tail” incident cases. To assess survival bias, we fitted a logistic regression for the cross-sectional association of the PGS-SZ and diabetes status at the baseline, in which we considered T2DM cases that occurred at early ages and compared the results to the main findings. Third, we re-ran the main models restricting the outcome definition to diagnosed T2DM cases only, as earlier ELSA studies did (Demakakos *et al*., 2012; Bell *et al.*, 2014).

Fourth, we tested the results’ sensitivity to the changes in the health and behaviour variables recorded at the subsequent follow-up. As the chosen statistical method was not extended to time-varying covariates, we could not test it directly. Instead, we compared the interval censoring method to the Cox model implemented in the R package survival (Therneau, 2022) for which we assigned event times to the middle of a respective interval. The results were similar in impact sizes and confidence intervals (CIs), so we fitted a Cox model with time-varying covariates. We included updated levels for BMI, triglycerides, HDL cholesterol, depressive symptoms, hypertension, stroke, cardiovascular disease, smoking and exercise regime. We then compared it to the Cox model with covariates fixed at the baseline.

## Results

### Sample

Our analytical sample included 5968 participants with a mean age of 64.9 (SD 9.2), and 2675 (44.8%) were men (Table 1). The average follow-up period in the present study was 8.7 years (SD 3.4); during this time, we identified 493 T2DM incident cases, 379 (76.9% of all cases) self-reported and 114 (23.1%) undiagnosed. Participants who developed T2DM had lower accumulated wealth and education level, higher BMI, higher prevalence of hypertension, higher triglyceride and lower HDL cholesterol, and were less likely to practice vigorous physical activities compared to those with no T2DM. Compared to other ELSA participants, our analytical sample had lower mean age, BMI, prevalence of hypertension and cardiovascular diseases and higher accumulated wealth. That was mainly due to the exclusion of the prevalent T2DM cases. Before that, the differences were more subdued, though higher hypertension rates and lower wealth persisted (Supplementary Tables 1 and 10, Supplemental digital content 1, http://links.lww.com/PG/A311).

**Table 1:** Baseline sample characteristics

Baseline characteristics	Type 2 diabetes by wave 8		Test statistics	*P* value
	No *N* = 5475 (91.7%)	Yes *N* = 493 (8.3%)		
	Mean (SD)/*n* (%)	Mean (SD)/*n* (%)	*t*(df)/*x*^2^(df)	
Length of follow-up, years	8.9 (3.4)	7.4 (3.3)	9.44 (5966)	<0.001
Age (years)	64.9 (9.3)	65.2 (8.6)	−0.81 (5966)	0.42
Sex				
Men	2431 (44.4)	244 (49.5)	4.74 (1)	0.030
Women	3044 (55.6)	249 (50.5)		
Relationship status				
Not married	1703 (31.1)	150 (30.4)	19.80 (1)	0.76
Married	3772 (68.9)	343 (69.6)		
BMI (kg/m^2^)	27.4 (4.6)	30.8 (5.3)	−15.35 (5966)	<0.001
Stroke				
No	5350 (97.7)	471 (95.5)	2.00 (1)	0.16
Yes	125 (2.3)	22 (4.5)		
History of hypertension				
No	3696 (67.5)	234 (47.5)	80.79 (1)	<0.001
Yes	1779 (32.5)	259 (52.5)		
History of cardiovascular disease				
No	4749 (86.7)	424 (86)	0.21 (1)	0.65
Yes	726 (13.3)	69 (14)		
Blood test				
Triglycerides (mmol/l)	1.7 (1.0)	2.2 (1.2)	−9.84 (5966)	<0.001
HDL cholesterol (mmol/l)	1.6 (0.4)	1.4 (0.3)	8.99 (5966)	<0.001
Severe depressive symptoms present				
No	4779 (87.3)	404 (81.9)	11.29 (1)	0.001
Yes	696 (12.7)	89 (18.1)		
Accumulated wealth				
Low	1641 (30)	200 (40.6)	30.2 (2)	<0.001
Intermediate	1818 (33.2)	163 (33.1)		
High	2016 (36.8)	130 (26.4)		
Education level				
Less than secondary	1672 (36.5)	188 (44.5)	17.89 (2)	<0.001
Secondary	2164 (47.3)	194 (46)		
Tertiary	740 (16.2)	40 (9.5)		
Smoking status				
Nonsmoker	4627 (84.8)	388 (79.2)	10.90 (1)	0.001
Smoker	827 (15.2)	102 (20.8)		
Exercise regime				
Light	256 (4.7)	30 (6.1)	19.8 (2)	<0.001
Moderate	3365 (61.5)	344 (69.8)		
Vigorous	1853 (33.9)	119 (24.1)		

### Polygenic risk score for schizophrenia and type 2 diabetes

**Fig. 2:**
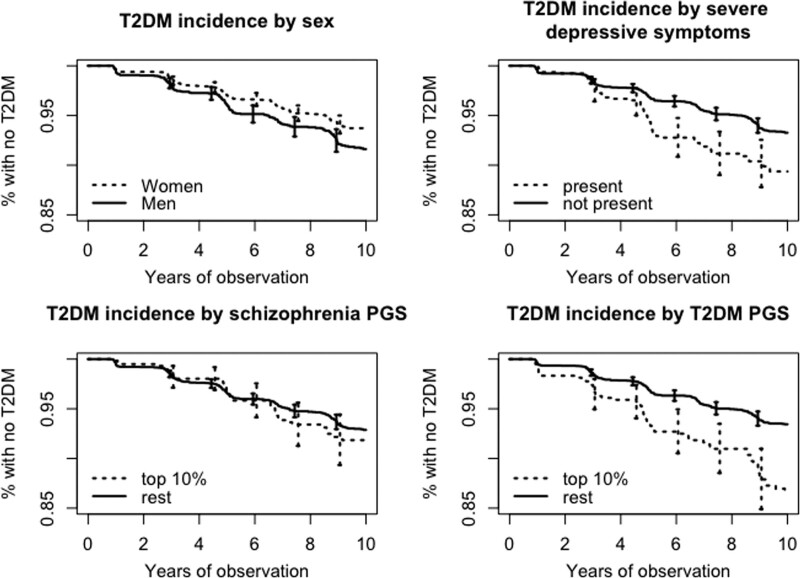
Survival curves for T2DM incidence by sex, exercise regime, wealth category, presence of severe depressive symptoms and polygenic scores for schizophrenia and T2DM. T2DM, type 2 diabetes with 95% confidence intervals.

We found no association between PGS-SZ and T2DM incidence during the 9-year follow-up period (Table 2). Estimated HRs for 1 SD increase in PGS-SZ were 1.01 (95% CI = 0.94–1.09) in Model 1 adjusted for age, sex and genetic ancestry and 1.04 (95% CI = 0.93–1.15) in the fully adjusted Model 2. Most of the other included risk factors were significant, including the less often included T2DM risk factors such as severe depressive symptoms (HR = 1.47; 95% CI, 1.08–1.77), or polygenic risk to T2DM (HR = 1.34; 95% CI, 1.21–1.47). Figure 2 plots cumulative T2DM incidence over the observation period by sex, presence of severe depressive symptoms, PGS-SZ and PGS-T2DM. In agreement with the regression analysis, participants grouped by the first three factors have visibly different T2DM survival curves, while survival curves for the individuals with high PGS-SZ and the rest look less dissimilar.

**Table 2:** Estimated hazard ratios for the type 2 diabetes incidence main study models

Estimated hazard ratios	Model 1	Model 2
	HR (95% CI)	HR (95% CI)
PGS-SZ (per 1 SD)	1.010 (0.932–1.095)	1.037 (0.933–1.152)
Age (per 10 years)	1.164 (1.047–1.294)**	1.210 (1.067–1.373)**
Sex: women	0.798 (0.668–0.954)*	0.763 (0.586–0.993)*
BMI (per 5 kg/m^2^)		1.572 (1.381–1.789)***
History of hypertension		1.632 (1.348–1.976)***
History of cardiovascular diseases		0.994 (0.721–1.372)
Severe depressive symptoms		1.352 (0.996–1.834)
Triglycerides (mmol/l)		1.112 (1.04–1.189)**
HDL cholesterol (mmol/l)		0.628 (0.385–1.025)
History of stroke		1.499 (0.972–2.312)
Current smoking		1.428 (1.095–1.864)**
Exercise: light		0.937 (0.606–1.451)
Exercise: vigorous		0.789 (0.602–1.036)
Education: low		1.448 (0.997–2.102)
Education: medium		1.342 (0.940–1.918)
Wealth: medium		1.021 (0.769–1.354)
Wealth: low		1.172 (0.894–1.537)
PGS-T2DM (per 1 SD)		1.335 (1.214–1.467)***

Model 1 adjusted for age, sex, genetic ancestry and schizophrenia polygenic score.

Model 2 adjusted for age, sex, genetic ancestry and schizophrenia polygenic score, BMI, hypertension, cardiovascular diseases, stroke, present severe depressive symptoms, blood triglycerides and HDL (mmol/l), current smoking (yes/no), exercise (light/moderate/vigorous; baseline level = moderate), wealth (low/medium/high; baseline level is “high”), education (low/medium/high, baseline level is “high”), polygenic score for T2DM.

CI, confidence interval, HR, hazard ratio, PGS-SZ, polygenic risk score for schizophrenia, T2DM, type 2 diabetes.

*P value <0.05 and above 0.01; **P value <0.01 and above 0.0001; ***P value <0.001.

### Sensitivity analyses

All sensitivity analyses yielded similar results, and the T2DM – PGS-SZ association remained statistically insignificant. In particular, restricting the sample to participants with complete records or changing the outcome definition to diagnosed T2DM cases had little impact on the estimated HRs, including the one for PGS-SZ. Cross-sectional analysis showed that baseline T2DM was not associated with PGS-SZ (odds ratio 1.03; 95% CI, 0.94–1.12 adjusted for sex, age and ancestry). Detailed results can be found in the Supplementary materials, Tables 5–8, Supplemental digital content 1, http://links.lww.com/PG/A311.

## Discussion

We aimed to investigate whether aggregated polygenic risk for schizophrenia is associated with the onset of T2DM during an average follow-up of 9 years in a sample of older adults without schizophrenia diagnosis residing in the UK. Our hypothesis was that intrinsic biological mechanisms underlying both diseases would manifest in an elevated T2DM incidence rate in people with high polygenic load to schizophrenia, which we tested using longitudinal regression analysis. Employing a sample without schizophrenia cases was a way to limit environmental factors associated with the hardships of living with this mental disorder (such as antipsychotic medication side effects, or low social support), while polygenic risk scores enabled us to quantify schizophrenia genetic risk in such a sample. We further adjusted for socio-economic and behavioural variables to single out the impact of the genetic factors. To our knowledge, this is the first study to examine this relationship in a representative sample of undiagnosed adults.

### Ascertainment of the outcome measure

Our T2DM outcome included self-reported T2DM cases diagnosed by a clinician and undiagnosed cases established by the blood tests. This translated into an observed 8.3% T2DM incidence in our sample over the 9 years, or 9.5 cases per 1000 person-years for the total incidence, and 7.3 per 1000 person-years for diagnosed cases only, consistent with other developed countries’ rates (Au *et al.*, 2014; Bell *et al*., 2014; Forouhi and Wareham, 2019), and estimates that a quarter of diabetes cases remain undiagnosed (Huang *et al.*, 2021). Further, we assumed that all diabetes cases were of type 2, however, the number of misspecified cases is estimated to be very low. In the UK, 94 out of 100 existing diabetes cases are of type 2 (NHS Digital, 2021), and the risk of T2DM increases with age (Pal *et al.*, 2021), while more than half of type 1 are diagnosed before 40 years (Rogers *et al.*, 2017), it is unlikely that more than 3% (15) of the observed 493 diabetes onsets were misspecified in our sample of adults aged 50 and above.

### Main findings in the context of previous research

The nexus of schizophrenia diagnosis and T2DM onset among adults is supported by the epidemiological (Frank *et al*., 2015; Annamalai *et al*., 2017; Pillinger *et al*., 2017; Rajkumar *et al*., 2017) and genetic research (Hackinger *et al*., 2018; So *et al*., 2019). Shared biological and genetic factors underlying T2DM and schizophrenia are also thought to be involved in the onset of these diseases (Lin and Shuldiner, 2010; Mizuki *et al*., 2020); however, we did not observe a significant association between polygenic predisposition to schizophrenia and T2DM in our sample.

This result is consistent with several other works such as a recent meta-analysis of the familial risk of glucose dysregulation and schizophrenia (Misiak *et al.*, 2020), Mendelian Randomization studies reporting no causal relationship of schizophrenia to T2DM, or T2DM to schizophrenia (Li *et al*., 2018; Polimanti *et al*., 2018; Aoki *et al*., 2022), or LDSR analysis showing a negative T2DM-schizophrenia correlation as opposed to an expected positive (Perry *et al*., 2022). Aoki and colleagues (Aoki *et al*., 2022) have even concluded that metabolic dysregulation in schizophrenia was likely to be due to schizophrenia environmental factors such as lifestyle habits, poor living conditions and antipsychotic medication.

However, we would not dismiss the supportive evidence and highlight the fact that even though our results point to a negligible impact of schizophrenia genetic factors on T2DM onset in the general population, they do not imply that such association is weak in people with schizophrenia due to potential GxE and ExE factors (Chung and Miller, 2019). For example, GxE studies found that genetic risk to schizophrenia may underly an increased sensitivity to metabolic stress (Brunelin *et al.*, 2008) or to psychological stress due to childhood adversities (Guloksuz *et al.*, 2019), both of which increase T2DM chances (Hackett and Steptoe, 2017). A compounding effect of the environmental factors (ExE) can be inferred from a positive correlation between the T2DM risk and the duration of psychotic illness (Philippe *et al.*, 2005). Thus, given persistent evidence of the first psychotic episode being a stressful event for the metabolic system (Pillinger *et al*., 2017), schizophrenia onset may mark a qualitative change in the body metabolism, which is absent in a sample of undiagnosed adults. Larger studies involving a sufficient number of diagnosed and undiagnosed adults could help quantify such effects.

Secondly, it is feasible that PGS-SZ's impact on T2DM incidence in adults undiagnosed with schizophrenia is smaller than what we could have detected in the cohort of six thousand adults. In this case, we can estimate an upper bound of the association strength using the results of the power calculations which suggested that an association with a HR of 1.14 or higher would have been detected, which is relatively low compared to the impact of other risk factors (Table 2). Therefore, if our finding is false negative, it is likely that 1 SD in PGS-SZ implies a lower than 14% increase in the T2DM hazard rate. Assuming the PRS in a population would be in the [−2SD, +2SD] range, most susceptible undiagnosed people would have less than a 30% increase in the instantaneous risk of T2DM compared to a median person.

Finally, while discussing the possibility of a false negative result, we should mention PGS limitations. PGS is a convenient tool that projects complex genetic architecture on a single axis realigned with the propensity of present schizophrenia (Wray *et al*., 2014), but such simplicity may come at a cost. Schizophrenia genetic variants responsible for metabolic abnormalities may have low weights in the constructing equation of PGS-SZ, making PGS-SZ ineffective in predicting T2DM risks. Interestingly, PGS-T2DM have been linked to the onset of psychosis (Perry *et al.*, 2020), which may indicate that PGS-T2DM better captures common genetic variants of the two diseases. Further, it is possible that previous studies focused on shared genetics, but less on the direction of their impact. While genes such as TCF7L2, TNF, APOE and BDNF-related genes can heighten T2DM and schizophrenia risk (Alkelai *et al.*, 2012; Mizuki *et al*., 2020; Perry *et al*., 2022), other genes may have opposing impacts and lead to inconsistent and biased LDSR findings (Perry *et al*., 2022). This effect may explain the negative genetic correlation between BMI and schizophrenia found by many (Bulik-Sullivan *et al*., 2015; Ikeda *et al.*, 2018; Aoki *et al*., 2022). Finally, genetic associations of metabolic traits and schizophrenia have been found sex- and age-dependent, with a stronger negative correlation in older age (Hübel *et al.*, 2019), and could have contributed to the negative findings in our sample of older adults.

### Strengths and weaknesses

The strength of this analysis is the employment of a representative sample of the English older adult population, which meant we investigated the association across a population-wide range of polygenic scores. The comprehensive list of the participants’ characteristics in the ELSA study allowed control of the health-related, socio-economic and behavioural variables, assessing the PGS-SZ and T2DM relationship on various levels. We addressed information bias from missing values by employing multiple imputations and complete case analyses, and bias due to undiagnosed T2DM outcomes by the respective sensitivity analyses. We catered for the uncertainty in the T2DM onset date by employing interval censoring, which has not been done yet in ELSA diabetes studies. The sample included people over 50, and as T2DM incidence tends to increase with age (Nichols *et al.*, 2015), that could have been beneficial for the sample’s statistical power. Our sensitivity analysis of the prevalent T2DM cases showed that people who developed T2DM before the baseline had similar T2DM-PGS-SZ relationships as those who developed T2DM during the observation period, suggesting limited survival bias.

There are several limitations of our study. First, we did not test any specific biological pathways or traits in which schizophrenia-related polygenic risk manifests. Second, it is feasible that PGSs utilized in the present study, having encompassed hundreds to thousands of common variants, accumulated noise which might have masked the genuine associations (Vilhjálmsson *et al.*, 2015). Third, information bias could be present due to undiagnosed or unreported cases of schizophrenia and other self-reported health-related covariates. Fourth, potential participation bias should be acknowledged, as our sample was slightly biased towards individuals with higher socio-economic status, similar to other genetic studies (Schoeler *et al.*, 2023). Extrapolation to younger populations should be made cautiously. Finally, worth noting the limited generalizability of our results to non-European populations, as underlying GWASs used predominantly European samples.

### Conclusion

Our results provide alternative evidence suggestive of the low contribution of the intrinsic biological mechanisms driven by the polygenic risk of schizophrenia on a future T2DM onset. By undertaking a quantitative approach we could estimate a 30% population-wide upper limit on aggregated polygenic risk to schizophrenia’s impact on the instantaneous T2DM risk, compared to a median risk in the population. Nevertheless, we do not exclude that schizophrenia-related environmental factors play a confounding or intermediating role in the clinical association of T2DM and schizophrenia, which we could not test in our setting, or that PGS-SZ did not fully represent schizophrenia-related genetic risk underlying T2DM development leading to a negative finding. Further research is needed.

## Acknowledgements

The English Longitudinal Study of Ageing is funded by the National Institute on Aging (RO1AG7644) and by a consortium of UK government departments coordinated by the National Institute for Health Research (NIHR). O.A. is further funded by an NIHR Post-Doctoral Fellowship (PDF-2018-11-ST2-020). D.S. is funded by the King’s College London BHI PhD studentship. A.R. and D.St. are part-funded by the NIHR Biomedical Research Centre at South London and Maudsley NHS Foundation Trust and King’s College London. A.R. is further part-supported by Health Data Research UK, an initiative funded by UK Research and Innovation, Department of Health and Social Care (England) and the devolved administrations, and leading medical research charities. The views expressed in this publication are those of the authors and not necessarily those of KCL, NHS, or NIHR. The funding organizations had no role in the design and conduct of the study; collection, management, analysis and interpretation of the data; preparation, review, or approval of the manuscript; and decision to submit the manuscript for publication.

### Conflicts of interest

There are no conflicts of interest.

## Supplementary Material


