# Supplementary material for: Schizophrenia polygenic risk score and type 2 diabetes onset in older adults with no schizophrenia diagnosis
Source: Psychiatr Genet. 2023 Jul 4;33(5):191–201. doi: 10.1097/YPG.0000000000000349 (PMC10501355; doi:10.1097/YPG.0000000000000349)
Supplement: Supplementary file 1 [file pg-33-191-s001.pdf]

Schizophrenia polygenic risk score and type 2 diabetes onset in older adults with no schizophrenia diagnosis.

Diana Shamsutdinova, MSc[1], Olesya Ajnakina, PhD[1,2], Angus Roberts, PhD[1], Daniel Stahl, PhD[1]

Supplementary materials

1. **Supplementary Table 1.** Characteristics of the ELSA participants included and excluded from the current analytical sample
2. **Supplementary Table 2.** Missing values at baseline
3. **Supplementary table 3.** Distribution of the variables before and after imputation
4. **Supplementary table 4.** Characteristics of participants with diagnosed (self-reported) and undiagnosed diabetes incidence
5. **Supplementary table 5.** Results of the complete cases analysis
6. **Supplementary Table 6.** Sensitivity analysis of introducing time-varying covariates and updating their values at the next medical follow-up
7. **Supplementary table 7**. Association of the T2DM at baseline and schizophrenia polygenic risk score. Results from the cross-sectional analysis using logistic regression and risk factors of the main study models
8. **Supplementary table 8.** Sensitivity analysis of changing the definition of the outcome to include diagnosed T2DM cases only compared to the main analysis which combined diagnosed and undiagnosed cases
9. **Supplementary table 9.** Power calculations

Supplementary Table 1. Characteristics of the ELSA participants included and excluded from the current analytical sample.

Baseline characteristics	ELSA participants N=13946	Excluded N=7978 (57.2%)	Included N=5968 (42.8%)	Test statistics	
	**Mean (SD) / n (%)**	**Mean (SD) / n (%)**	**Mean (SD) / n (%)**	***t*(df)/x²(df)**	**P**
**Age (years)**	65.1(10.3)	65.3(11)	64.9(9.2)	1.96(13944)	0.0504
**Gender**					
Men	6351(45.5)	3676(46.1)	2675(44.8)	2.17(1)	0.1411
Women	7595(54.5)	4302(53.9)	3293(55.2)		
**Relationship status**					
Not married	4203(33.1)	2350(34.9)	1853(31.0)	385.84(1)	<0.001
Married	8500(66.9)	4385(65.1)	4115(69.0)		
**Body mass index (kg/m2)**	28.1(5.1)	28.6(5.5)	27.6(4.7)	9.23(13944)	<0.001
**History of hypertension**					
No	7724(61.6)	3794(57.8)	3930(65.9)	85.69(1)	<0.001
Yes	4808(38.4)	2770(42.2)	2038(34.1)		
**History of cardiovascular disease**					
No	10461(83.5)	5288(80.6)	5173(86.7)	84.83(1)	<0.001
Yes	2071(16.5)	1276(19.4)	795(13.3)		
**Severe depressive symptom present**					
No	8401(84.7)	3218(81.5)	5183(86.8)	52.28(1)	<0.001
Yes	1515(15.3)	730(18.5)	785(13.2)		
**Accumulated wealth**					
Low	4382(35.4)	2541(39.6)	1841(30.8)	120.43(2)	<0.001
Intermediate	4022(32.5)	2041(31.8)	1981(33.2)		
High	3983(32.2)	1837(28.6)	2146(36.0)		
**Education level**					
Less than secondary	4574 (40.8)	2714 (43.6)	1860(37.2)	47.67(2)	<0.001
Secondary	5028 (44.8)	2670 (42.9)	2358(47.2)		
Tertiary	1620 (14.4)	840 (13.5)	780(15.6)		
**Smoking status**					
Non-smoker	10557(83.3)	5542(82.4)	5015(84.4)	8.74(1)	0.0031
Smoker	2112(16.7)	1183(17.6)	929(15.6)		
**Exercise regime**					
Light or none	1197(9.6)	911(14.0)	286(4.8)	385.84(2)	<0.001
Moderate	7799(62.5)	4090(62.9)	3709(62.2)		
Vigorous	3475(27.9)	1503(23.1)	1972(33)		

df, degrees of freedom; SD, standard deviation; P-p-value

Supplementary Table 2. Missing values at baseline

Missing values	***At baseline***
Characteristic	***Missing***<br>***N (%, out of the total sample of 5968)***
PGS-SZ	0
Age	0
Gender	0
Body mass index (kg/m2)	354 (5.9%)
History of hypertension	0
History of cardiovascular disease	0
Assessment of severe depressive symptoms	0
History of stroke	0
Triglycerides	396 (6.6%)
HDL	398 (6.7%)
Exercise regime	1 (0.0%)
Current smoking	24 (0.4%)

Supplementary table 3. Distribution of imputed variables before and after imputation

N of imputed set	Smoking	Exercise regime			Triglyceride				HDL			
	% yes	Light or none (%)	moderate (%)	vigorous(%)	mean	sd	0.25% quantile	75% quantile	mean	sd	25% quantile	75% quantile
0	15.6%	4.8%	62.2%	33.1%	1.75	1.06	1.50	2.10	1.56	0.39	1.50	1.80
1	15.6%	4..8%	62.2%	33.0%	1.75	1.07	1.50	2.20	1.56	0.39	1.50	1.80
2	15.6%	4.8%	62.2%	33.1%	1.75	1.07	1.50	2.10	1.56	0.39	1.50	1.80
3	15.6%	4.8%	62.2%	33.0%	1.75	1.06	1.50	2.10	1.56	0.39	1.50	1.80
4	15.6%	4.8%	62.2%	33.1%	1.75	1.06	1.50	2.20	1.56	0.39	1.50	1.80
5	15.6%	4.8%	62.2%	33.0%	1.76	1.10	1.50	2.10	1.56	0.39	1.50	1.80
6	15.6%	4.8%	62.2%	33.0%	1.75	1.06	1.50	2.10	1.56	0.39	1.50	1.80
7	15.6%	4.8%	62.2%	33.0%	1.75	1.07	1.50	2.20	1.56	0.39	1.50	1.80
8	15.6%	4.8%	62.2%	33.0%	1.76	1.08	1.50	2.20	1.56	0.39	1.50	1.80
9	15.6%	4.8%	62.2%	33.0%	1.76	1.07	1.50	2.20	1.56	0.39	1.50	1.80
10	15.6%	4.8%	62.2%	33.0%	1.75	1.10	1.50	2.10	1.56	0.39	1.50	1.80
11	15.7%	4.8%	62.2%	33.1%	1.76	1.08	1.50	2.20	1.56	0.40	1.50	1.80
12	15.6%	4.8%	62.2%	33.0%	1.75	1.07	1.50	2.10	1.56	0.39	1.50	1.80
13	15.6%	4.8%	62.2%	33.0%	1.75	1.07	1.50	2.10	1.56	0.39	1.50	1.80
14	15.6%	4.8%	62.2%	33.0%	1.75	1.07	1.50	2.10	1.56	0.39	1.50	1.80
15	15.6%	4.8%	62.2%	33.0%	1.75	1.09	1.50	2.10	1.56	0.39	1.50	1.80
16	15.6%	4.8%	62.2%	33.0%	1.75	1.07	1.50	2.20	1.56	0.39	1.50	1.80
17	15.6%	4.8%	62.2%	33.0%	1.75	1.07	1.50	2.10	1.56	0.39	1.50	1.80
18	15.6%	4.8%	62.2%	33.1%	1.75	1.07	1.50	2.10	1.56	0.39	1.50	1.80
19	15.6%	4.8%	62.2%	33.0%	1.75	1.06	1.50	2.10	1.56	0.40	1.50	1.80
20	15.6%	4.8%	62.2%	33.0%	1.75	1.07	1.50	2.10	1.56	0.39	1.50	1.80

Supplementary table 4. Characteristics of participants with diagnosed (self-reported) and undiagnosed diabetes incidence

**Baseline characteristics**	**T2DM incidences**	**Diagnosed or Undiagnosed case**		**Test statistics**	
	**N=493**	**Diagnosed N=379 (76.9%)**	**Undiagnosed N=114 (23.1%)**		
	Mean (SD) / n (%)	Mean (SD) / n (%)	Mean (SD) / n (%)	*t*(df)/x^2(df)	P
Length of follow-up, years	6.6(3)	6.6(3.1)	10.2(2.5)	-11.27(491)	<0.001
Age (years)	65.2(8.6)	64.8(8.6)	66.7(8.7)	-2.03(491)	0.0425
Gender					
Men	244(49.5)	191(50.4)	53(46.5)	0.53(1)	0.4647
Women	249(50.5)	188(49.6)	61(53.5)		
Relationship status					
not married	150(30.4)	120(31.7)	30(26.3)	1.93(1)	0.2770
married	343(69.6)	259(68.3)	84(73.7)		
Body mass index (kg/m2)	30.8(5.3)	30.8(5.1)	30.7(6)	0.17(491)	0.8676
Stroke					
No	471(95.5)	364(96)	107(93.9)	0.98(1)	0.1573
Yes	22(4.5)	15(4)	7(6.1)		
History of hypertension					
No	234(47.5)	180(47.5)	54(47.4)	<0.01(1)	0.9810
Yes	259(52.5)	199(52.5)	60(52.6)		
History of cardiovascular disease					
No	424(86)	326(86)	98(86)	<0.01(1)	0.9890
Yes	69(14)	53(14)	16(14)		
Blood test					
Triglycerides	2.2(1.2)	2.3(1.2)	2.0(1.0)	1.96(491)	0.0510
HDL	1.4(0.3)	1.4(0.3)	1.4(0.3)	-1.22(491)	0.2235
Severe depressive symptom present					
No	404(81.9)	310(81.8)	94(82.5)	0.03(1)	0.8720
Yes	89(18.1)	69(18.2)	20(17.5)		
Accumulated wealth					
Low	200(40.6)	158(41.7)	42(36.8)	0.94(2)	0.6249
Intermediate	163(33.1)	124(32.7)	39(34.2)		
High	130(26.4)	97(25.6)	33(28.9)		
Education level					
Less than secondary	188(44.5)	137(42.4)	51(51.5)	4.96(2)	0.0838
Secondary	194(46.0)	158(48.9)	36(36.4)		
Tertiary	40(9.5)	28(8.7)	12(12.1)		
Smoking status					
Non-smoker	388(79.2)	302(80.1)	86(76.1)	0.84(1)	0.3583
Smoker	102(20.8)	75(19.9)	27(23.9)		
Exercise regime					
Light or none	30(6.1)	23(6.1)	7(6.1)	1.93(2)	0.3817
Moderate	344(69.8)	259(68.3)	85(74.6)		
Vigorous	119(24.1)	97(25.6)	22(19.3)		

Supplementary table 5. Results of the complete cases analysis

Results of the main analyses using records of participants with no missing data required for each of the models. Model 1 was based on the sample of 5968 individuals (there were no missing data in Model A's covariates), Model 2 - 4459 (74.7% of the main analytical sample).

Estimated hazard ratios	*Model 1*	*Model 1a*	*Model 1b*	*Model 1c*	*Model 2*
	**HR (95%CI)**	**HR (95%CI)**	**HR (95%CI)**	**HR (95%CI)**	**HR (95%CI)**
PGS-SZ (per 1 sd)	1.010(0.905,1.128)	1.018(0.958,1.082)	1.017(0.922,1.122)	1.008(0.904,1.124)	1.024(0.903,1.161)
Age (per 10y)	1.164(1.08,1.254)***	1.235(1.085,1.407)**	1.272(1.161,1.393)***	1.215(1.064,1.387)**	1.24(1.1,1.397)***
Gender _women	0.798(0.663,0.961)*	0.853(0.695,1.046)	0.815(0.626,1.06)	0.773(0.603,0.990)*	0.753(0.602,0.943)*
BMI (per 5kg/m2)		1.618(1.425,1.838)***	1.633(1.522,1.753)***	1.626(1.494,1.769)***	1.630(1.468,1.811)***
History of hypertension		1.594(1.311,1.938)***	1.603(1.348,1.906)***	1.589(1.294,1.95)***	1.540(1.226,1.935)***
History of CVD		1.082(0.846,1.384)	1.051(0.815,1.356)	1.032(0.658,1.619)	1.020(0.736,1.414)
Present severe depressive symptoms		1.491(1.177,1.89)***	1.405(1.072,1.841)*	1.344(1.047,1.725)*	1.353(1.05,1.743)*
Triglycerides (mmol/l)		1.175(1.097,1.258)***	1.157(1.08,1.24)***	1.149(1.07,1.234)***	1.147(1.079,1.220)***
HDL Cholesterol (mmol/l)		0.548(0.379,0.793)**	0.578(0.368,0.906)*	0.539(0.367,0.791)**	0.568(0.372,0.869)**
History of stroke		1.720(1.12,2.641)*	1.538(1.081,2.187)*	1.631(0.902,2.948)	1.649(0.995,2.735)
Current smoking			1.642(1.33,2.028)***	1.559(1.108,2.195)*	1.493(1.068,2.086)*
Exercise_light			1.049(0.673,1.636)	1.064(0.537,2.108)	1.026(0.647,1.627)
Exercise_vigorous			0.809(0.645,1.014)	0.881(0.73,1.063)	0.877(0.731,1.051)
Education level_ low				1.556(1.072,2.260)*	1.481(1.022,2.146)*
Education level _medium				1.343(0.914,1.973)	1.282(0.903,1.819)
Wealth_medium				1.003(0.751,1.338)	0.991(0.745,1.317)
Wealth_low				1.091(0.743,1.603)	1.073(0.784,1.469)
PGS-T2DM (per 1 sd)					1.303(1.151,1.475)***

*** for p-value <0.001, ** for p-value <0.01 and above 0.0001, * for p-value <0.05 and above 0.01

Key: All models are semi-parametric proportionate hazard models with interval censoring implemented in the IcenReg R package.
Model 1 included age, gender, and schizophrenia polygenic score.
Models 1a, 1b, and 1c added physical health, lifestyle factors, and socioeconomic variables in a stepwise manner.
Finally, Model 2 included age, gender, and schizophrenia polygenic score (with 4 genetic principal components to adjust for ancestry), and BMI, history of hypertension, history of cardiovascular disease, history of stroke, present depression, baseline blood triglycerides and HDL cholesterol levels (mmol/l); and current smoking(yes/no), exercise (light/moderate/vigorous; baseline level = moderate); accumulated wealth (low/medium/high; baseline level is "high"), education (low/medium/high, baseline level is "high"), polygenic score for T2DM.

Supplementary Table 6. Sensitivity analysis of introducing time-varying covariates and updating their values at the next medical follow-up

We compared IcenReg semi-parametric proportionate hazards model accounting for interval censoring but not for time-varying covariates, corresponding Cox model assuming the time of event in the middle of the interval, and Cox model accounting for the time-varying covariates.

Estimated hazard ratios for Model E	Interval censoring<br><br>time-invariant covariates (baseline only)<br>HR (95%CI)	Cox model<br><br>time-invariant covariates (baseline only)<br>HR (95%CI)	Cox model<br>time-varying covariates (baseline and updated at the next medical visit)<br>HR (95%CI)
**PGS-SZ (per 1 sd)**	1.037(0.933,1.152)	1.034(0.935,1.144)	1.033(0.938,1.137)
**Age (per 10y)**	1.21(1.067,1.373)**	1.229(1.09,1.386)***	1.302(1.149,1.474)***
**Gender _women**	0.763(0.586,0.993)*	0.741(0.57,0.963)*	0.787(0.617,1.005)
**BMI (per 5kg/m2)**	1.572(1.381,1.789)***	1.598(1.415,1.804)***	1.505(1.357,1.67)***
**History of hypertension**	1.632(1.348,1.976)***	1.677(1.367,2.057)***	1.406(1.153,1.714)***
**History of CVD**	0.994(0.721,1.372)	0.998(0.755,1.32)	0.763(0.574,1.015)
**Present severe depressive symptoms**	1.352(0.996,1.834)	1.384(1.051,1.822)*	1.409(1.082,1.834)*
**Triglycerides (mmol/l)**	1.112(1.04,1.189)**	1.115(1.049,1.184)***	1.177(1.091,1.27)***
**HDL Cholesterol (mmol/l)**	0.628(0.385,1.025)	0.623(0.391,0.991)*	0.537(0.349,0.826)**
**History of stroke**	1.499(0.972,2.312)	1.555(0.967,2.502)	1.119(0.699,1.789)
**Current smoking**	1.428(1.095,1.864)**	1.484(1.155,1.908)**	1.862(1.428,2.427)***
**Exercise_light**	0.937(0.606,1.451)	0.967(0.64,1.463)	0.671(0.43,1.047)
**Exercise_vigorous**	0.789(0.602,1.036)	0.776(0.599,1.005)	0.821(0.648,1.04)
**Education level _ low**	1.448(0.997,2.102)	1.467(0.995,2.163)	1.324(0.826,2.122)
**Education level _medium**	1.342(0.94,1.918)	1.352(0.93,1.965)	1.218(0.776,1.913)
**Wealth_medium**	1.021(0.769,1.354)	1.018(0.77,1.345)	1.087(0.835,1.414)
**Wealth_low**	1.172(0.894,1.537)	1.193(0.915,1.555)	1.268(0.958,1.677)
**PGS-T2DM (per 1 sd)**	1.335(1.214,1.467)***	1.357(1.235,1.490)***	1.34(1.214,1.479)***

Supplementary table 7. Association of the T2DM at baseline and schizophrenia polygenic risk score. Results from the cross-sectional analysis using logistic regression and risk factors of the study models

	**Model 1**	**Model 2**
**Odds Ratio estimate**	**1.0021**	**1.0042**
**p-value**	**0.5211**	**0.2522**

The sample for this analysis contained individuals with complete data for the underlying risk factors used in each of the model.
Model 1: age, gender, and schizophrenia polygenic score (with 4 genetic principal components to adjust for ancestry).
Model 2: age, gender, and schizophrenia polygenic score (with 4 genetic principal components to adjust for ancestry), and BMI, history of hypertension, history of cardiovascular disease, history of stroke, present depression, baseline blood triglycerides and HDL cholesterol levels (mmol/l); and current smoking(yes/no), exercise (light/moderate/vigorous; baseline level = moderate); accumulated wealth (low/medium/high; baseline level is “high”), education (low/medium/high, baseline level is “high”), polygenic score for T2DM.
Results: We did not observe a statistically significant association between the T2DM diagnosis at the baseline and polygenic risk score for schizophrenia.

Supplementary table 8. Sensitivity analysis of changing the definition of the outcome to include diagnosed T2DM cases only compared to the main analysis which combined diagnosed and undiagnosed cases

Results for the most inclusive model (Model E) which comprised the full list of the covariates used in the study.

	Definition of the outcome	
	**Diagnosed cases only**	**Diagnosed and undiagnosed cases**
Estimated hazard ratios	**Model E, HR (95%CI)**	**Model E, HR (95%CI)**
PGS-SZ (per 1 sd)	**1.055(0.945,1.178)**	**1.037(0.933,1.152)**
Age (per 10y)	1.152(0.995,1.334)	1.210(1.067,1.373)**
Gender _women	0.772(0.592,1.006)	0.763(0.586,0.993)*
BMI (per 5kg/m2)	1.535(1.367,1.724)***	1.572(1.381,1.789)***
History of hypertension	1.623(1.291,2.040)***	1.632(1.348,1.976)***
History of CVD	0.982(0.691,1.395)	0.994(0.721,1.372)
Present severe depressive symptoms	1.361(1.001,1.851)*	1.352(0.996,1.834)
Triglycerides (mmol/l)	1.119(1.035,1.209)**	1.112(1.040,1.189)**
HDL Cholesterol (mmol/l)	0.565(0.345,0.927)*	0.628(0.385,1.025)
History of stroke	1.317(0.769,2.256)	1.499(0.972,2.312)
Current smoking	1.267(0.919,1.748)	1.428(1.095,1.864)**
Exercise_light	0.981(0.602,1.599)	0.937(0.606,1.451)
Exercise_vigorous	0.855(0.654,1.118)	0.789(0.602,1.036)
Education level _ low	1.387(0.719,2.674)	1.448(0.997,2.102)
Education level _medium	1.424(0.786,2.578)	1.342(0.94,1.918)
Wealth_medium	1.077(0.782,1.483)	1.021(0.769,1.354)
Wealth_low	1.333(0.98,1.814)	1.172(0.894,1.537)
PGS-T2DM (per 1 sd)	1.329(1.187,1.488)***	1.335(1.214,1.467)***

*** for p-value <0.001, ** for p-value <0.01 and above 0.0001, * for p-value <0.05 and above 0.01

Supplementary table 9. Power calculations

Sample size required to find the effect statistically significant with probability 80%, using the definition of a statistically significant level at p-value < 0.05

Estimated hazard ratio	*Required sample size, Model A*	*Required sample size, Model E*
1.05	37987	38222
1.06	26380	26543
1.07	19381	19501
1.08	14839	14931
1.09	11725	11797
1.105	9497	9556
1.12	7849	7897
1.13	6595	6636
1.14	**5620**	**5655**
1.15	4846	4876
1.16	4221	4247
1.22	2375	2389
1.65	380	383
2.72	95	96

Using R package "powerSurvEpi" , function ssizeEpiCont

R code

```
size_for_effectsize = function(df0, exposure = "sz20_", modelcov = covE, effectisizes = c(0.1,0.125, 0.15,0.2,0.5,1)){
  list_na = vector(mode = "double")
  for (i in seq(length(effectsizes))) {
    n = ssizeEpiCont(formula =  as.formula(paste(exposure, "~",   paste(modelcov, collapse = "+"), sep ="")),
                     dat = df0,    var.X1 = exposure,   var.failureFlag = 'outcome_d',     power = 0.80,
                     theta = exp(effectsizes[i]),         alpha = 0.05)["n"]
                   list_na[i] = n  }
 res = data.frame(estimated_effectsize = effectsizes, exp_effectsize = exp(effectsizes), n = unlist(list_na))
 return(res)
}
effectsizes = c(0.05, 0.06,0.07,0.08, 0.09, 0.1,0.11,0.12,0.13,0.14, 0.145,0.15,0.2,0.5,1)
size_for_effectsize(df, "sz20_", model_a_0, effectsizes)
size_for_effectsize(df, "sz20_", model_d_0, effectsizes)
```

Conclusion: in our analytical sample (N=5968) an association with the hazard ratio of 1.14/1 sd or above can be detected with probability 80% using the alpha level (p-value threshold) 0.05.

Supplementary table 10. Further analysis of the included and excluded participants into the analytical cohort of the study out of all ELSA participants.

In the Supplementary Table 1 we have compared the cohort of people in the final analytical sample ("included" group) versus all the rest ("excluded" group) for our analytical cohort. So, the "excluded" group contained prevalent T2DM cases that were excluded from our time-to-event analysis of the incident cases, along with the participants who they did not opt in for the genotype testing. The excluded group then had a disproportionally large number of people with T2DM at the baseline, which resulted in higher prevalence of T2DM related risk factors. To check the potential selection bias and loss of statistical power, it can be better to compare the initial cohorts, that is, the participants with the genetic data, and without. Those are less dissimilar, but there is still a higher share of people with hypertension, cardiovascular diseases, and depressive symptoms ("ELSA Participants, ALL" sub-table below).

We have further compared the in/out cohorts within the same wealth categories ("ELSA Participants, Low/Medium/High Wealth" sub-tables below). The differences in BMI, depressive symptoms, marriage status, age (in low and medium wealth groups), and smoking cease to be statistically significant. Hypertension prevalence stays elevated in the excluded group (in medium and high wealth), as well as the cardiovascular diseases (in all wealth categories), although we note that a prior cardiovascular disease were not significant in our fully stratified model.

The presented stratified comparisons show that the cohort differences are substantially lower once stratified by wealth, and therefore, these differences should have had a limited impact on our results, especially in the fully adjusted models.

ELSA Participants, ALL	Excluded from the analyses*	Included in the analyses*	All	p-value	t(df)/x²(df)
Sex (% female)	55.13%	53.82%	54.46%	0.12058	2.41
Hypertension (% yes)	40.54%	36.72%	38.37%	0.00001	19.04
Depressive symptoms (% yes)	18.93%	13.70%	15.28%	0	44.11
Cardiovascular diseases (% yes)	19.04%	14.62%	16.53%	0	43.49
Smoking (% yes)	17.54%	15.99%	16.67%	0.02082	5.34
Wealth (% high)	29.63%	34.86%	32.60%	0	38.58
Physical activity (% high)	23.67%	31.00%	27.86%	0	251.46
Married (% yes)	64.74%	15.55%	23.06%	0	21.17
Body mass index (mean)	28.35	27.91	28.06	0.00003	4.18
Age (mean)	64.94	65.3	65.12	0.03602	-2.1
Years of Education (mean)	13.42	14	13.75	0	-8.08

ELSA Participants, Low Wealth	Excluded*	Included*	All	p_value	test statistic
Sex (female)	58.0%	57.1%	57.5%	0.5183	0.42
Hypertention (yes)	45.5%	43.3%	44.3%	0.1340	2.25
Depression (yes)	26.4%	20.7%	22.6%	0	14.85
Cardiovascular diseases (yes)	23.9%	18.0%	20.6%	0	23.26
Smoking (yes)	23.5%	25.9%	24.8%	0.0701	3.28
Wealth (high)	low	low	low		
Physical activity (high)	14.7%	19.7%	17.5%	0	107.19
Married (yes)	48.6%	99.6%	99.8%	0.1392	2.19
Body mass index	28.8	28.6	28.7	0.2405	1.17
Age	67.0	66.4	66.7	0.0619	1.87
Years of Education	12.0	12.3	12.2	0	-3.62

ELSA Participants, Medium Wealth	Excluded*	Included*	All	p_value	test statistic
Sex (female)	55.9%	54.0%	54.8%	0.2405	1.38
Hypertention (yes)	41.3%	37.3%	39.1%	0.0123	6.27
Depression (yes)	14.6%	13.0%	13.5%	0.2556	1.29
Cardiovascular diseases (yes)	18.0%	14.4%	16.0%	0.0030	8.78
Smoking (yes)	15.3%	13.8%	14.4%	0.1887	1.73
Wealth (high)	medium	medium	medium		
Physical activity (high)	23.7%	31.1%	27.9%	0	63.30
Married (yes)	71.3%	99.1%	99.7%	0.7165	0.13
Body mass index	28.4	28.1	28.2	0.0851	1.72
Age	64.9	65.4	65.1	0.1325	-1.50
Years of Education	13.4	13.8	13.6	0	-3.43

ELSA Participants, High Wealth	Excluded*	Included*	All	p_value	test statistic
Sex (female)	52.9%	50.6%	51.5%	0.1580	1.99
Hypertention (yes)	34.6%	30.4%	32.0%	0.0063	7.46
Depression (yes)	10.2%	7.8%	8.4%	0.0398	4.23
Cardiovascular diseases (yes)	14.2%	12.1%	12.9%	0.0472	3.94
Smoking (yes)	9.1%	8.2%	8.5%	0.2763	1.19
Wealth	high	high	high		
Physical activity (high)	35.7%	41.6%	39.4%	0	37.25
Married (yes)	81.6%	2.9%	4.2%	0.5308	0.39
Body mass index	27.6	27.0	27.2	0.0020	3.09
Age	62.6	64.5	63.7	0	-6.70
Years of Education	15.6	15.8	15.7	0.1516	-1.43

* Included cohort is the initial cohort selected for the analyses before exclusion of the prevalent diabetes cases. Excluded are all the rest, that is, those, for whom there is no genetic information.
